# High‐dose corticosteroid use and risk of hospitalization for infection in patients treated with immune checkpoint inhibitors––A nationwide register‐based cohort study

**DOI:** 10.1002/cam4.4040

**Published:** 2021-06-08

**Authors:** Signe Sørup, Bianka Darvalics, Leo Russo, Dina Oksen, Francois‐Xavier Lamy, Patrice Verpillat, Khalil AA, Sørensen HT, Deirdre Cronin‐Fenton

**Affiliations:** ^1^ Department of Clinical Epidemiology Aarhus University Hospital Aarhus Denmark; ^2^ Worldwide Medical and Safety Pfizer Collegeville PA USA; ^3^ Global Epidemiology Merck KGaA Darmstadt Germany; ^4^ Department of Oncology Aarhus University Hospital Aarhus Denmark

**Keywords:** corticosteroid, hospitalization for infection, PD‐1 immune checkpoint inhibitor, PD‐L1 immune checkpoint inhibitor

## Abstract

High‐dose corticosteroids have been associated with increased risk of serious infection in patients with metastatic melanoma treated with immune checkpoint inhibitors targeting cytotoxic T‐lymphocyte antigen 4. This potential association needs to be examined further among patients with other cancer types and for other immune checkpoint inhibitors. We examined whether receipt of high‐dose corticosteroids was associated with increased rates of hospitalization for infection among 981 Danish renal, urothelial, and lung cancer patients followed from first administration of programmed death receptor 1 (PD‐1)/programmed death ligand 1 (PD‐L1) immune checkpoint inhibitors. Our cohort analysis was based on the information from national medical registries. During follow‐up, 522 patients (53.2%) initiated treatment with high‐dose corticosteroids and 317 patients (32.3%) experienced at least one hospitalization for infection. In analyses adjusted for age, sex, and previous use of chemotherapy/targeted therapy, initiation of high‐dose systemic corticosteroids was associated with increased rate of hospitalization for infections (hazard ratio (HR) = 2.96, 95% confidence interval (CI) = 2.41–3.65) even in patients not receiving any chemotherapy/targeted therapy (HR = 3.66, 95% CI = 2.25–5.96). Our findings showed that high‐dose corticosteroid initiation is associated with hospitalization for infection in patients treated with PD‐1/PD‐L1 immune checkpoint inhibitors. Clinicians and patients should be aware of this risk of infection when initiating treatment with high‐dose corticosteroids.

## Graphical abstract

High‐dose corticosteroids have been associated with increased risk of serious infection in metastatic melanoma patients treated with immune checkpoint inhibitors (ICIs) targeting cytotoxic T‐lymphocyte antigen 4. The present study adds that use of high‐dose corticosteroids is also associated with increased rate of hospitalization for infection among lung, kidney, and urothelial cancer patients treated with ICIs targeting programmed death receptor 1 or programmed death ligand 1 even in patients who did not receive chemotherapy/targeted therapy.

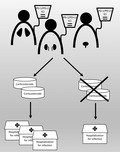

## 1 INTRODUCTION

Treatment with immune checkpoint inhibitors targeted against programmed cell death 1 (PD‐1) and programmed cell death ligand 1 (PD‐L1) have demonstrated prolonged survival for some types of cancer.^1^ PD‐1/PD‐L1 immune checkpoint inhibitors block tumor‐related downregulation of the immune system, thereby enhancing antitumor immunity.^2^ PD‐1/PD‐L1 immune checkpoint inhibitor treatment has been associated with adverse events and immune‐related adverse events such as hypothyroidism, hyperthyroidism, diarrhea, vitiligo, and pneumonitis,^3^ which can be managed with high‐dose corticosteroids.^4^, ^5^, ^6^ Del Castillo and colleagues found that high‐dose corticosteroid use was associated with serious infection (odds ratio [OR] = 7.71, 95% confidence interval [CI] = 3.71–16.18) in a bivariate analysis of patients with metastatic melanoma treated with immune checkpoint inhibitors (mainly targeting cytotoxic T‐lymphocyte antigen 4).^7^ Fujita and colleagues studied non‐small cell lung cancer patients treated with the PD‐1 immune checkpoint inhibitor, nivolumab, and found that 53% of patients with infections during follow‐up had received corticosteroids during or after nivolumab administration compared with 45% among those without infections during follow‐up (*p* value = 0.42 [no association measures or confidence intervals were reported]).^8^ In both studies, it was not indicated whether corticosteroids were given before or after the infection limiting the possibility to ensure the temporality between exposure and outcome. However, Fujita and colleagues also reported that 31% of patients with infections during follow‐up had received corticosteroids before administration of nivolumab compared with 19% among those without infections during follow‐up (*p* value = 0.14 [no association measures or confidence intervals reported]).^8^ Likewise, Karam and colleagues reported that infection‐free survival was similar between those with and without corticosteroid/immunosuppressant use before initiation of immune checkpoint inhibitors (primarily targeting PD‐1/PD‐L1) in cancer patients.^9^ These analyses of corticosteroid use before initiation of immune checkpoint inhibitors do not account for corticosteroids given after initiation of immune checkpoint inhibitors. Therefore, we examined whether the use of high‐dose corticosteroids after first administration of PD‐1/PD‐L1 immune checkpoint inhibitors was associated with an increased rate of hospitalization for infection among renal, urothelial, and lung cancer patients treated with PD‐1/PD‐L1 immune checkpoint inhibitors.

## 2 METHODS

We performed a nationwide medical registry‐based cohort study of Danish cancer patients. First, we selected all patients diagnosed with American Joint Committee on Cancer^10^ stage III or IV renal cell carcinoma, stage III or IV non‐small cell lung cancer, or stage II, III, or IV urothelial carcinoma between 1 January 2013 and 31 December 2017, according to the Danish Cancer Registry which registers all cancers diagnosed in Denmark.^11^ Second, we identified patients registered with treatment codes for PD‐1/PD‐L1 immune checkpoint inhibitors during hospital contacts in the Danish National Patient Registry^12^ covering all Danish hospitals between 1 January 2013 and 1 June 2018. We followed patients from the first date of treatment with PD‐1/PD‐L1 immune checkpoint inhibitors (index date) until 1 year (365 days) after the first registration of treatment with these agents, death, emigration, diagnosis of a new primary cancer, or 31 December 2018 (last date with available data), whichever came first.

High‐dose corticosteroid was defined as a redemption of prescriptions at community pharmacies with the anatomic therapeutic classification code H02AB [systemic glucocorticoids] and ≥25 mg active substance per pill registered in the Danish National Prescription Registry.^13^ This definition was decided based on clinical experience to focus our study on high‐dose corticosteroids and exclude low‐dose corticosteroids with less active substance per pill.

The outcome was in‐patient hospitalizations with any primary or secondary *International Classification of Diseases*, *Tenth Revision* (ICD‐10) diagnosis code for infections.

### 2.1 Statistical methods

We used a Cox proportional hazards model to compute hazard ratios (HRs) of hospitalizations for infections, comparing time after first redemption of high‐dose corticosteroids with no use of high‐dose corticosteroids (time‐varying exposure). Time since first administration of PD‐1/PD‐L1 immune checkpoint inhibitors was the underlying time scale. We used the Andersen–Gill model to account for recurrent events and included robust standard errors for estimating 95% CIs.^14^ We performed both unadjusted analyses and analyses adjusted for sex, age, and previous chemotherapy/targeted therapy. We evaluated effect modification by sex and previous chemotherapy/targeted therapy. We performed two sensitivity analyses: 1) censoring at first administration of chemotherapy/targeted therapy during follow‐up and 2) excluding and censoring on obstructive lung disease and brain metastases that might be treated with high‐dose corticosteroids.

We used SAS version 9.4 for analyses.

## 3 RESULTS

The study included 981 patients treated with PD‐1/PD‐L1 immune checkpoint inhibitors. They were followed until death (N = 484; 48.3%), 1 year of follow‐up (N = 409; 41.7%), 31 December 2018 (N = 88; 9.0%), new primary cancer (N < 10), or emigration (N < 5).

The median number of administrations of PD‐1/PD‐L1 immune checkpoint inhibitors was 4 (interquartile range [IQR] = 2–12; Table 1). During follow‐up, 522 patients (53.2%) redeemed high‐dose corticosteroids at a median of 3.5 months after PD‐1/PD‐L1 immune checkpoint inhibitor initiation (IQR = 2.0–6.4 months; Table 1).

**TABLE 1:** Description of Danish renal, urothelial, and lung cancer patients with at least one administered PD‐1/PD‐L1 immune checkpoint inhibitor

Variable	Renal cell carcinoma (N = 70)	Urothelial carcinoma (N = 59)	Non‐small cell lung cancer (N = 852)	All cancers combined (N = 981)
Stage at diagnosis^a^, N (%)				
Stage II	Not included	11 (18.6%)	Not included	11 (1.1%)
Stage III	<25	<10	317 (37.2%)	343 (35.0%)
Stage IV	49 (70.0%)	24 (40.7%)	520 (61.0%)	593 (60.4%)
Undefined stage^b^	<5	<20	15 (1.8%)	34 (3.5%)
Male, N (%)	47 (67.1%)	33 (55.9%)	438 (51.4%)	518 (52.8%)
Age at inclusion (years), Median (IQR)	62.7 (53.9–68.2)	70.3 (65.1–75.7)	68.6 (62.2–73.6)	68.4 (61.9–73.5)
Received chemotherapy and/or targeted therapy before first administration of PD−1/PD‐L1 immune checkpoint inhibitor, N (%)	45 (64.3%)	51(86.4%)	637 (74.8%)	733 (74.7%)
Year of first administration of PD−1/PD‐L1 immune checkpoint inhibitors (=year of inclusion in the study), N (%)				
2015	<10	<5	33 (3.9%)	41 (4.2%)
2016	<15	<5	226 (26.5%)	241 (24.6%)
2017	39 (55.7%)	29 (49.2%)	486 (57.0%)	554 (56.5%)
2018	15 (21.4%)	23 (39.0%)	107 (12.6%)	145 (14.8%)
Follow‐up time (months)^c^, Median (IQR)	12.0 (7.9–12.0)	8.8 (5.0–12.0)	10.1 (5.0–12.0)	10.1 (5.2–12.0)
Received chemotherapy and/or targeted therapy during follow‐up, N (%)	32 (45.7%)	15 (25.4%)	248 (29.1%)	295 (30.1%)
Number of administrations of PD−1/PD‐L1 immune checkpoint inhibitors during follow‐up^d^, Median (IQR)	5 (2–14)	4 (1–12)	4 (1–11)	4 (2–12)
Type of PD−1/PD‐L1 immune check point inhibitor^e^, N (%)				
PD−1 immune check point inhibitor	65 (92.9%)	46 (78%)	844 (99.1%)	955 (97.3%)
PD‐L1 immune check point inhibitor	5 (7.1%)	15 (25.4%)	9 (1.1%)	29 (3%)
Received high‐dose corticosteroids during follow‐up, N (%)	33 (47.1%)	32 (54.2%)	457 (53.6%)	522 (53.2%)
Time from first administration of PD−1/PD‐L1 immune checkpoint inhibitors to first redemption of high‐dose corticosteroids^f^ (months), Median (IQR)	3.6 (1.8–6.3)	3.5 (1.9–6.5)	3.4 (2.0–6.3)	3.5 (2.0–6.4)

Note

In accordance with guidelines issued by the Danish Health Data Authority, we were not permitted to report cell counts with fewer than five observations. As well, the cell count may not be identifiable based on counts in the other cells; for this reason we also report “N<10” etc. in some cells.

Abbreviations: IQR, interquartile range; N, number; PD‐1, programmed death receptor 1; PD‐L1, programmed death ligand 1.

^a^ Stage is defined according to the 7th Edition of the American Joint Committee on Cancer Staging Manual.

^b^ Some information on T, N, or M codes was missing, but based on available information a given patient had at least stage II (urothelial carcinoma) or stage III (renal cell carcinoma and non‐small cell lung cancer) cancer.

^c^ Time from the first date of treatment with PD‐1/PD‐L1 immune checkpoint inhibitors until the first of the following events: 1 year (365 days) after the first registration of treatment with PD‐1/PD‐L1 checkpoint inhibitors in the Danish National Patient Registry, death, registration of a new type of primary cancer in the Danish Cancer Registry, emigration, or 31 December 2018.

^d^ Number of days for which treatment codes for PD‐1/PD‐L1 immune checkpoint inhibitors were registered in the Danish National Patient Registry.

^e^ Give the number and the proportion of patients getting the specified type at least once. Each patient might get both types during follow‐up.

^f^ Among patients receiving high‐dose corticosteroids.

Overall, 317 patients (32.3%) experienced at least one hospitalization for infection. The most frequent infections were pneumonia (48.5%), unspecified bacterial infections (13.3%), sepsis (11.0%), and urinary tract infections (10.5%, Table S1). The adjusted HR for hospitalizations for infections was 2.96 (95% CI=2.41–3.65), comparing high‐dose corticosteroid use with no use (Table 2). Among patients who had not previously received chemotherapy/targeted therapy, the adjusted HR was 4.02 (95% CI = 2.72–5.95; Table 2) but reduced to 3.66 (95% CI = 2.25–5.96; Table 3) when censoring at administration of chemotherapy/targeted therapy during follow‐up. There were no major differences by sex (Table 2). Excluding and censoring on diagnosis of chronic obstructive lung disease and brain metastases did not substantially alter the results (Table S2).

**TABLE 2:** Association between high‐dose corticosteroid use and hospitalizations for infection

Patient group and exposure	Incidence rate per 100 person‐years (N hospitalizations for infections/PYRs)	HR^a^ (95% CI)	Adjusted HR^b^ (95% CI)
Renal cell carcinoma (N = 70)			
No corticosteroid	26.8 (11/41)	1 (ref)	1 (ref)
Corticosteroid	69.5 (11/16)	2.35 (0.89–6.18)	2.53 (0.90–7.10)
Urothelial carcinoma (N = 59)			
No corticosteroid	76.0 (23/30)	1 (ref)	1 (ref)
Corticosteroid	254.9 (25/10)	5.01 (2.58–9.74)	4.53 (2.33–8.80)
Non‐small cell lung cancer (N = 852)			
No corticosteroid	54.8 (254/464)	1 (ref)	1 (ref)
Corticosteroid	127.5 (174/137)	2.86 (2.29–3.58)	2.84 (2.27–3.56)
All cancers combined (N = 981)			
*Overall*			
No corticosteroid	53.8 (288/535)	1 (ref)	1 (ref)
Corticosteroid	129.5 (210/162)	2.98 (2.42–3.67)	2.96 (2.41–3.65)
*Effect modification by sex*			
Females			
No corticosteroid	51.8 (135/260)	1 (ref)	1 (ref)
Corticosteroid	115.2 (88/76)	2.75 (2.05–3.69)	2.74 (2.04–3.68)
Males			
No corticosteroid	55.8 (153/274)	1 (ref)	1 (ref)
Corticosteroid	142.3 (122/86)	3.17 (2.43–4.13)	3.16 (2.42–4.12)
*P interaction*		0.45	0.46
*Effect modification by previous chemotherapy*/*targeted therapy* ^c^			
Previous chemotherapy/targeted therapy			
No corticosteroid	60.2 (233/387)	1 (ref)	1 (ref)
Corticosteroid	130.7 (156/119)	2.70 (2.14–3.42)	2.71 (2.14–3.42)
No previous chemotherapy/targeted therapy			
No corticosteroid	37.2 (55/148)	1 (ref)	1 (ref)
Corticosteroid	126.1 (54/43)	4.08 (2.76–6.03)	4.02 (2.72–5.95)
*P interaction*		0.07	0.08

Abbreviations: CI, confidence interval; HR, hazard ratio; N, number; PD‐1, programmed death receptor 1; PD‐L1, programmed death ligand 1; PYRs, person‐years at risk.

^a^ Estimated by Cox proportional hazards model with time since first administration of PD‐1/PD‐L1 immune checkpoint inhibitors as the underlying time scale. Use of high‐dose corticosteroid is a time‐varying exposure; thus, all person time occurring before the first redemption of a prescription for high‐dose corticosteroid is included in “no corticosteroid ” and all person time occurring after the first redemption of a prescription for high‐dose corticosteroid is included in “corticosteroid”.

^b^ Same model as under footnote a, but additionally adjusted for age (categorical), sex (only models without effect modification by sex), and chemotherapy/targeted therapy before first administration of PD‐1/PD‐L1 immune checkpoint inhibitors(only models without effect modification by chemotherapy/targeted therapy).

^c^ Patients who have received chemotherapy/targeted therapy before first administration of PD‐1/PD‐L1 immune checkpoint inhibitors are in the “Previous chemotherapy/targeted therapy” group.

**TABLE 3:** Association between high‐dose corticosteroid use and hospitalizations for infection when censoring at first administration of chemotherapy/targeted therapy during follow‐up.

Patient group and exposure	Incidence rate per 100 person‐years (N hospitalizations for infections/PYRs)	HR^a^ (95% CI)	Adjusted HR^b^ (95% CI)
All cancers combined (N = 981)			
*Overall*			
No corticosteroid	50.5 (231/457)	1 (ref)	1 (ref)
Corticosteroid	119.5 (136/114)	3.21 (2.51–4.12)	3.23 (2.52–4.14)
*Effect modification by sex*			
Females			
No corticosteroid	49.5 (111/224)	1 (ref)	1 (ref)
Corticosteroid	100.2 (56/56)	2.74 (1.92–3.92)	2.75 (1.92–3.94)
Males			
No corticosteroid	51.5 (120/233)	1 (ref)	1 (ref)
Corticosteroid	138.2 (80/58)	3.65 (2.67–4.99)	3.67 (2.68–5.03)
*P interaction*		0.21	0.21
*Effect modification by previous chemotherapy/targeted therapy* ^c^			
Previous chemotherapy/targeted therapy			
No corticosteroid	56.9 (186/327)	1 (ref)	1 (ref)
Corticosteroid	127.6 (104/81)	3.07 (2.33–4.05)	3.11 (2.36–4.10)
No previous chemotherapy/targeted therapy			
No corticosteroid	34.5 (45/130)	1 (ref)	1 (ref)
Corticosteroid	99.0 (32/32)	3.72 (2.29–6.04)	3.66 (2.25–5.96)
*P interaction*		0.49	0.55

Abbreviations: CI, confidence interval; HR, hazard ratio; N, number; PD‐1, programmed death receptor 1; PD‐L1, programmed death ligand 1; PYRs, person‐years at risk.

^a^ Estimated by Cox proportional hazards model with time since first administration of PD‐1/PD‐L1 immune checkpoint inhibitors as the underlying time scale. Use of high‐dose corticosteroid is a time‐varying exposure; thus, all person time occurring before the first redemption of a prescription for high‐dose corticosteroid is included in “nocorticosteroid ” and all person time occurring after the first redemption of aprescription for high‐dose corticosteroid is included in “corticosteroid”.

^b^ Same model as under footnote a, but additionally adjusted for age (categorical), sex (only models without effect modification by sex), and chemotherapy/targeted therapy before first administration of PD‐1/PD‐L1 immune checkpoint inhibitors(only models without effect modification by chemotherapy/targeted therapy).

^c^ Patients who have received chemotherapy/targeted therapy before first administration of PD‐1/PD‐L1 immune checkpoint inhibitors are in the “Previouschemotherapy/targeted therapy” group.

## 4 DISCUSSION

Our findings showed that a third of patients treated with PD‐1/PD‐L1 immune checkpoint inhibitors were hospitalized for infection and that the rate of hospitalization for infection was nearly three times higher if initiation of treatment with a high‐dose corticosteroid occurred.

This study was based on nationwide registries from a setting with free‐of‐charge, universal access to health care with virtually complete coverage and follow‐up.^15^, ^16^ However, using national registries has the limitation that not all potentially relevant confounders are included. Furthermore, we had no information on the reason for corticosteroid prescription and the duration of corticosteroid use.

The results of our study confirmed the previous observation by Del Castillo and colleagues of increased risk of serious infections associated with corticosteroid use in metastatic melanoma patients treated with immune checkpoint inhibitors in a bivariate analysis.^7^ We also note Fujita and colleagues’ study of non‐small cell lung cancer patients treated with nivolumab, which found that 53% of patients with infections during follow‐up had received corticosteroids during or after nivolumab administration compared with 45% among those without infections during follow‐up.^8^ In contrast to these previous studies, we performed a multivariate Cox regression analysis with corticosteroid use as a time‐varying variable ensuring that only person‐time after initiation of corticosteroid use contributed to the exposed group.

The studies by Fujita and colleagues^8^ and Karam and colleagues^9^ reported the bivariate analyses of corticosteroid use before initiation of treatment with immune checkpoint inhibitors and infections after initiation of treatment with immune checkpoint inhibitors. In contrast, we did not include corticosteroid prescriptions before the first administration of PD‐1/PD‐L1 immune checkpoint inhibitors. However, we performed a sensitivity analysis excluding and censoring patients who could likely receive high‐dose corticosteroids for other indications than immune‐related adverse events (chronic obstructive lung disease and brain metastases). This sensitivity analysis showed similar results to the main analysis.

The proportion of patients experiencing infections was 32.3% in our study compared with 7.3% in the study by Del Castillo and colleagues,^7^ 19% in the study by Fujita and colleagues,^8^ and 18% in the study by Karam and colleagues.^9^ These discrepancies could be related to differences in the definition of infection. Del Castillo and colleagues identified serious infections by reviewing microbiological and imaging results for indications of infection followed by review of medical records for subsequent hospitalization or treatment with parenteral antimicrobials.^7^ Fujita and colleagues and Karam and colleagues based their definition on the use of antimicrobial agents.^8^, ^9^ In contrast, we identified hospitalization for infection using diagnosis codes recorded in the Danish National Patient Registry capturing clinical diagnoses. A previous study found that a diagnosis code for infection had a positive predictive value of 98% (95% CI = 96%–99%) among Danish hospitalized cancer patients.^17^ However, differences could also be related to different settings and that the present study mainly included non‐small cell lung cancer patients treated with PD‐1 immune checkpoint inhibitors, while for example, Del Castillo and colleagues^7^ included melanoma patients mainly treated with cytotoxic T‐lymphocyte antigen 4 immune checkpoint inhibitors.

Nearly half of the infections were registered as pneumonia in our study. However, it is possible that clinicians miscoded the immune‐related adverse event, pneumonitis, as pneumonia. Such miscoding would be most likely in patients who had not yet been diagnosed with immune‐related adverse events and thereby have a lower likelihood of treatment with high‐dose corticosteroid. Thus, such miscoding would contribute most hospitalizations for infections in the time periods without exposure to corticosteroid and thereby lead to an underestimation of the association between corticosteroids and hospitalizations for infections.

Both our study and that conducted by Del Castillo and colleagues^7^ showed that use of high‐dose corticosteroids was associated with increased risk of infections requiring treatment among several types of cancer patients who were prescribed different immune checkpoint inhibitors. However, our study adds that this association also occurs among patients who did not receive chemotherapy/targeted therapy before or after initiation of treatment with PD‐1/PD‐L1 immune checkpoint inhibitors. However, our findings may be attributable to confounding by indication whereby the conditions requiring treatment with high‐dose corticosteroids are associated with the risk of infection. Still, it is important that clinicians and patients be aware of this risk of infection when initiating treatment with high‐dose corticosteroids and the benefit of such treatment needs to be carefully assessed. Guidelines on how to manage immune‐related adverse events in patients treated with immune checkpoint inhibitors have been published.^5^, ^6^

### 4.1 Conclusion

We showed that treatment with high‐dose corticosteroids was associated with hospitalization for infection in renal, urothelial, and lung cancer patients treated with PD‐1/PD‐L1 immune checkpoint inhibitors. However, further research into this area is needed.

## CONFLICT OF INTEREST

S.S., B.D., H.T.S., and D.C. report no personal conflict of interest. L.R. is a full‐time employee of Pfizer, Inc. D.O., F.L., and P.V. are full‐time employees of Merck KGaA, Darmstadt, Germany. A.A.K. reports honoraria from AstraZeneca A/S for speaking at a Nordic‐Baltic scientific meeting, "1ST Nordic/Baltic multidisciplinary scientific exchange meeting on treatment of inoperable stage III NSCLC patients” (2019).

## AUTHORS CONTRIBUTION

Study concept and design: S.S., B.D., and D.C. Acquisition, analysis, or interpretation of data: S.S., B.D., L.R., D.O., F.L., P.V., A.A.K., H.T.S., and D.C. Statistical analysis: B.D. Drafting of the manuscript: S.S. Critical revision of the manuscript for important intellectual content: B.D., L.R., D.O., F.L., P.V., A.A.K., H.T.S., and D.C.

## ETHICAL STATEMENT

Under Danish law, ethical permission from the regional ethical committee is not required for registry‐based research. This study followed the EU General Data Protection Regulation. Data were obtained from the Danish Health Data Authority.

## Supporting information

Supplementary Material

## Data Availability

Due to Danish data protection rules we are not allowed to share the data. However, the study is based on data from national Danish registers. Access to this register data can be obtained by applying to the Danish Health Data Authority.
