# Supplementary material for: High‐dose corticosteroid use and risk of hospitalization for infection in patients treated with immune checkpoint inhibitors––A nationwide register‐based cohort study
Source: Cancer Med. 2021 Jun 8;10(14):4957–63. doi: 10.1002/cam4.4040 (PMC8290247; doi:10.1002/cam4.4040)
Supplement: Supplementary file 1 — Supplementary Material [file CAM4-10-4957-s001.doc]

**Supplementary Appendix: High-dose corticosteroid use and risk of hospitalization for infection in patients treated with immune checkpoint inhibitors –A nationwide register-based cohort study**

**CONTENT**

**Table S1.** Distribution of type of infection registered during the hospitalization with any type of infections

**Table S2.** Association between high-dose corticosteroid use and hospitalizations when excluding patients with a diagnosis of chronic obstructive pulmonary disease or brain metastasis

| **Table S1.** Distribution of type of infection registered during the hospitalization with any type of infections | |
| --- | --- |
| **Type of hospital-diagnosed infectious diseases** | **Number of registrations, N (%)** |
| Pneumonia | 189 (48.5%) |
| Other respiratory infections | 13 (3.3%) |
| Sepsis | 43 (11.0%) |
| Urinary tract infections | 41 (10.5%) |
| Gastrointestinal infections | 9 (2.3%) |
| Intra-abdominal infections | 8 (2.1%) |
| Skin infections | 7 (1.8%) |
| Bacterial infections, unspecified | 52 (13.3%) |
| Fungal infections | 9 (2.3%) |
| Other infections | 19 (4.9%) |
| Note: If a patient was registered with ICD-10 diagnosis codes from several groups of infections during the same hospitalization, the hospitalization was counted in both groups. The percentage is the percentage among all registrations. | |

| **Table S2.** Association between high-dose corticosteroid use and hospitalizations when excluding 162 patients who have a diagnosis of chronic obstructive pulmonary disease or brain metastasis before initiation of follow-up and censor at first diagnosis of chronic obstructive pulmonary disease[ICD-10 code J44] and brain metastases [ICD-10 code C79.3]during follow-up. | | | |
| --- | --- | --- | --- |
| **Patient group and exposure** | **Incidence rate per 100 person- years (N hospitalizations for infections/PYRs)** | **HR^a^ (95% CI)** | **Adjusted HR^b^ (95% CI)** |
| **All cancers combined** (N=819) |  |  |  |
| ***Overall*** |  |  |  |
| No corticosteroid | 52.8 (234/444) | 1 (ref) | 1 (ref) |
| Corticosteroid | 127.4 (145/114) | 2.93 (2.31 to 3.72) | 2.94 (2.31 to 3.73) |
| ***Effect modification by sex*** |  |  |  |
| Females |  |  |  |
| No corticosteroid | 53.1 (110/207) | 1 (ref) | 1 (ref) |
| Corticosteroid | 100.1 (55/55) | 2.28 (1.60 to 3.26) | 2.27 (1.59 to 3.25) |
| Males |  |  |  |
| No corticosteroid | 52.4 (124/236) | 1 (ref) | 1 (ref) |
| Corticosteroid | 153.0 (90/59) | 3.55 (2.64 to 4.79) | 3.56 (2.64 to 4.80) |
| *P interaction* |  | 0.05 | 0.05 |
| ***Effect modification by previous chemotherapy/targeted therapy^c^*** |  |  |  |
| Previous chemotherapy/targeted therapy |  |  |  |
| No corticosteroid | 59.2 (189/319) | 1 (ref) | 1 (ref) |
| Corticosteroid | 128.5 (106/83) | 2.64 (2.01 to 3.46) | 2.66 (2.03 to 3.49) |
| No previous chemotherapy/targeted therapy |  |  |  |
| No corticosteroid | 36.2 (45/124) | 1 (ref) | 1 (ref) |
| Corticosteroid | 124.7 (39/31) | 4.07 (2.59 to 6.38) | 4.03 (2.57 to 6.33) |
| *P interaction* |  | 0.10 | 0.11 |
| Abbreviations: CI=confidence interval; HR=hazard ratio; N=number; PD-1=programmed death receptor 1; PD-L1=programmed death ligand 1; PYRs=person-years at risk. ^a^ Estimated by Cox proportional hazards model with time since first administration of PD-1/PD-L1 immune checkpoint inhibitors as the underlying time scale. Exposure is high-dose corticosteroid as a time-varying variable meaning that all person time occurring before the first redemption of a prescription for high-dose corticosteroid is included in “no corticosteroid”, all person time occurring after the first redemption of a prescription for high-dose corticosteroid is included in “corticosteroid”.  ^b^ Same model as under footnote a, but additionally adjusted for age (categorical), sex (only models without effect modification by sex), and chemotherapy/targeted therapy before first administration of PD-1/PD-L1 immune checkpoint inhibitors(only models without effect modification by chemotherapy/targeted therapy)  ^c^ Effect modification according to if chemotherapy/targeted therapy had been administered before first administration of PD-1/PD-L1 immune checkpoint inhibitors | | | |
